# Prognostic value of GRACE score for in-hospital and 6 months outcomes after non-ST elevation acute coronary syndrome

**DOI:** 10.1186/s43044-021-00146-9

**Published:** 2021-03-06

**Authors:** Dileep Kumar, Arti Ashok, Tahir Saghir, Naveedullah Khan, Bashir Ahmed Solangi, Tariq Ahmed, Musa Karim, Khadijah Abid, Reeta Bai, Rekha Kumari, Hitesh Kumar

**Affiliations:** 1 National Institute of Cardiovascular Diseases (NICVD), Karachi, Pakistan; 2 College of Physicians and Surgeons Pakistan (CPSP), Karachi, Pakistan; 3 Dow University of Health Sciences, Karachi, Pakistan; 4 Government of Sindh, Karachi, Pakistan

**Keywords:** GRACE risk score, Acute coronary syndrome, Non-ST elevation acute coronary syndrome, In-hospital mortality, Predictor, Risk factors

## Abstract

**Background:**

The aim of this study was to determine the predictive value of the Global Registry of Acute Coronary Events (GRACE) score for predicting in-hospital and 6 months mortality after non-ST elevation acute coronary syndrome (NSTE-ACS).

**Results:**

In this observational study, 300 patients with NSTE-ACS of age more than 30 years were included; 16 patients died during the hospital stay (5.3%). Of 284 patients at 6 months assessment, 10 patients died (3.5%), 240 survived (84.5%), and 34 were lost to follow-up (12%) respectively. In high risk category, 10.5% of the patients died within hospital stay and 11.8% died within 6 months (*p* = 0.001 and *p* = 0.013). In univariate analysis, gender, diabetes mellitus, family history, smoking, and GRACE score were significantly associated with in-hospital mortality whereas age, obesity, dyslipidemia, and GRACE were significantly associated with 6 months mortality. After adjustment, diabetes mellitus, family history, and GRACE score remained significantly associated with in-hospital mortality (*p* ≤ 0.05) and age remained significantly associated with 6 months mortality.

**Conclusion:**

GRACE risk score has good predictive value for the prediction of in-hospital mortality and 6 months mortality among patients with NSTE-ACS.

## Background

Acute coronary syndrome (ACS) is a syndrome caused by decreased blood flow in the coronary arteries. The clinical characteristics of ACS, including ST segment elevation of myocardial infarction (STEMI), non-STEMI, and unstable angina, is known to be widespread causes for disability and mortality [1]. Life-saving therapies for ACS patients are strongly dependent on early and prompt identification of signs and symptoms, whereas atypical appearance of ACS symptoms may lead to delayed diagnosis, delayed care, less evidence-based approaches, and increased morbidity and mortality [2].

Accurate stratification of risk factors and diagnostic evaluation are of the highest significance not just for primary prevention but even for the prevention of repeated coronary ischemia or infarction attacks [3]. For patients with confirmed ACS diagnosis, various scoring systems may be used in order to differentiate patients in the coronary care unit that benefit more from the treatments. The risk scores such as the thrombolysis in myocardial infarction (TIMI), platelet glycoprotein IIb/IIIa in unstable angina: receptor suppression using integrilin (PURSUIT), fast revascularization in instability in coronary disease (FRISC), and Global Registry of Acute Coronary Events (GRACE) are well validated in this regard. But none of the scoring systems were used to identify the ACS in the emergency room [4]. A huge number of patients with chest pain due to factors other than ACS were not assessed in these studies. Currently, there is actually no evidence based risk stratification and guidelines for these patients [4].

There are many risk scores for ACS risk stratification [5]. GRACE [The Global Registry of Acute Coronary Events] score is one of the score that was developed to identify patients in the coronary care unit or emergency department at the greatest risk of adverse events after ACS. It has been observed that the odds of in-hospital mortality have increased significantly with increase in GRACE score [4]. The GRACE score was validated in various databases and c-statistics of the GRACE score was estimated to be 0.83 in the original database [4]. The parameters of the GRACE score (range 2 to 372) are heart rate, age, systolic blood pressure, cardiac arrest, Killip class, ST segment deviation, serum creatinine, and cardiac biomarker status [5].

In Pakistan, coronary diseases have been the leading causes of morbidity and mortality [6]. The goal of the current research is therefore to evaluate the predictive importance of the GRACE score for predicting in-hospital and 6-month non-ST acute coronary syndrome (NSTE-ACS) mortality.

## Methods

It was an observational study conducted at the department of a tertiary care cardiac center of Karachi, Pakistan, from August 2019 to August 2020. Sample size was estimated by using OpenEpi online sample size calculator; using 95% confidence level, 5.6% margin of error, and statistics for GRACE score ≥ 217 for predicting in-hospital mortality as 39.7% [7]. Sample size for the study was 294 ≈ 300 patients. Patients with NSTE-ACS of age more than 30 years of either gender were included in the study. Patients with prior history of cardiac-related surgery or intervention and ST elevation myocardial infarction and patients who refuse to give consent were excluded from the study.

The study was conducted after the approval of ethical review committee of the institution. Verbal informed consent was obtained from all the eligible participants. Data regarding baseline characteristics, positive family history, smoking status, hypertension, diabetes mellitus, and obesity were collected from all the eligible participants on pre-designed questionnaire. GRACE score was calculated based on age, heart rate, systolic blood pressure, Killip class, cardiac arrest, ST segment deviation, serum creatinine, and initial cardiac biomarker status [4]. The tertiles of risk categories used were as follows: for in-hospital mortality, low risk for GRACE score ≤ 108, intermediate risk for GRACE score between 109 and 140, and high risk for GRACE score ≥ 141. All patients were managed as per the institutional protocol and guidelines. Patients were followed for 6 months (telephonic follow-up) and study outcomes [i.e., mortality (in-hospital and 6 months mortality] were recorded.

Data analysis was done using SPSS version 25. Mean and SD were computed for numeric variables. Frequency and percentage were computed for categorical variables. Fisher exact test was applied to compare risk categories with in-hospital and 6 months mortality. Receiver operating characteristic (ROC) curves were used to determine the predictive accuracy of the GRACE scores regarding in-hospital and 6 months mortality. Prediction was deemed significant when the area under the ROC curve (AUC) was statistically different from 0.5. Univariate logistic regression was performed to identify the significant predictors of in-hospital and 6 months mortality. Variables which were significant in univariate analysis at 20% level of significance were moved to single multivariate model. A *p* value ≤ 0.05 was considered as statistically significant.

## Results

The mean age of the study subjects was estimated as 58.04 ± 10.70 years (range 32–95 years). Most of the patients were males (76.7%) and 23.3% were females. Hypertension was the most frequent risk factor (84.7%), followed by diabetes (42.3%). Only 9% of the patients were obese and 27.3% were smokers. Out of 300 patients, 11% had positive family history of coronary artery disease in first degree relative.

The mean GRACE score was 120.19 ± 33.17 (range 44–233). Most of the patients had low risk (*n* = 119, 39.7%), followed by moderate risk (*n* = 105, 35%) and high risk (*n* = 76, 25.3%). Of 300 patients, 16 patients died during the hospital stay (5.3%). Of 284 patients at 6 months assessment, 10 patients died (3.5%), 240 survived (84.5%), and 34 were lost to follow-up (12%) respectively. In high risk category, 10.5% of the patients died within hospital stay and 11.8% died within 6 months (*p* = 0.001 and *p* = 0.013) (Table 1).

**Table 1:** Estimation of mortality with respect to risk categories based on GRACE score

Risk categories based on GRACE score	Low risk	Moderate risk	High risk	***p*** value
**In-hospital mortality**	0	7 (6.7%)	9 (11.8%)	0.001*
**Six months mortality**	1 (0.9%)	3 (3.4%)	6 (10.5%)	0.013*

*significant at 5%

**Fig. 1:**
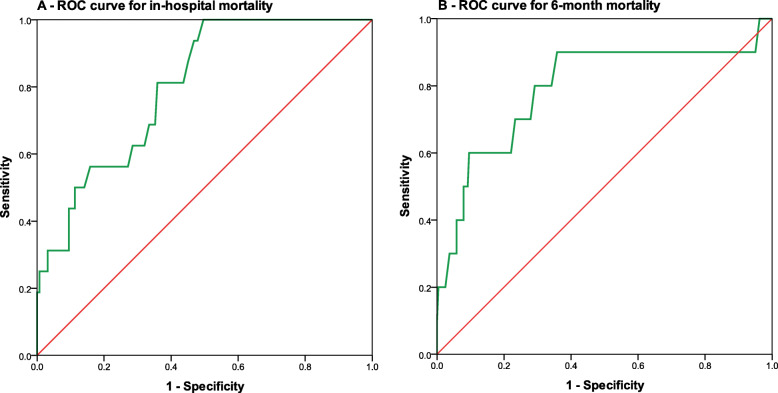
ROC curve for in-hospital mortality (**a**) and 6 months mortality (**b**)

Figure 1a shows the receiver operating characteristic [ROC] for GRACE score for the prediction of in-hospital mortality [AUC = 0.80, 95% CI 0.71–0.89; *p* = 0.001]. The best cutoff point for GRACE score derived from ROC is 190.5 ≈ 120 which gives sensitivity of 93.8% and specificity of 53.2%. Figure 1b shows the receiver operating characteristic [ROC] for GRACE score for the prediction of 6 months mortality [AUC = 0.792, 95% CI 0.619–0.964; *p* = 0.002]. The best cutoff point for GRACE score derived from ROC is 130.5 ≈ 131 which gives sensitivity of 80% and specificity of 70.8%.

In univariate analysis, gender, diabetes mellitus, family history, smoking, and GRACE score were significantly associated with in-hospital mortality. Variables which were significant in univariate analysis at *p* ≤ 0.20 were moved into multivariate analysis. After adjustment, diabetes mellitus, family history, and GRACE score remained significantly associated with in-hospital mortality (*p* ≤ 0.05) (Table 2).

**Table 2:** Univariate analysis and multivariate analysis for in-hospital mortality

Predictors	Univariate analysis				Multivariate analysis			
	OR	95% CI for OR		***p*** value	AOR	95% CI for AOR		***p*** value
**Age** in years	1.03	0.982	1.08	0.228				
**Gender (female)**	2.728	0.977	7.617	0.055	2.041	0.635	6.560	0.231
**Hypertension**	2.824	0.364	21.92	0.321				
**Diabetes mellitus**	3.186	1.078	9.413	0.036*	4.473	1.224	16.350	0.023*
**Family history**	2.931	0.887	9.683	0.078	8.517	1.829	39.667	0.006*
**Smoking**	0.364	0.081	1.639	0.188	0.444	0.082	2.398	0.345
**Obesity**	0.662	0.084	5.211	0.695				
**Dyslipidemia**	0.779	0.171	3.549	0.747				
**GRACE score (≥ 120)**	17.03	2.22	130.66	0.006*	22.905	2.744	191.172	0.001*

*significant at 5%

In univariate analysis, age, obesity, dyslipidemia, and GRACE were significantly associated with 6 months mortality. Variables which were significant in univariate analysis at *p* ≤ 0.20 were moved into multivariate analysis. After adjustment, only age remained significantly associated with 6 months mortality (*p* ≤ 0.05) (Table 3).

**Table 3:** Univariate analysis and multivariate analysis for 6 months mortality

Predictors	Univariate analysis				Multivariate analysis			
	OR	95% CI for OR		***p*** value	AOR	95% CI for AOR		***p*** value
**Age** in years	1.152	1.066	1.245	0.001*	1.113	1.023	1.210	0.012*
**Gender (female)**	0.841	0.173	4.076	0.83				
**Hypertension**	0.729	0.149	3.57	0.697				
**Diabetes mellitus**	1	0.275	3.637	0.999				
**Family history**	1.048	0.127	8.649	0.965				
**Smoking**	0.659	0.136	3.184	0.604				
**Obesity**	3.083	0.609	15.615	0.174	2.619	0.393	17.471	0.320
**Dyslipidemia**	2.51	0.62	10.171	0.197	4.828	0.732	31.834	0.102
**GRACE score (≥ 130)**	9.151	1.897	44.147	0.006*	6.472	0.888	47.152	0.065

*significant at 5%

## Discussion

The GRACE score is globally recommended for the risk stratification of hospitalized patients with NSTE-ACS, so that they can obtain evidence-based care according to their risk group for potential ischemic incidents in future [3, 8, 9]. Large GRACE registry of patients with ACS used GRACE risk score for the prediction of myocardial infraction or death within hospital stay or at 6 months [10]. Although multiple researches have tested the validity of the GRACE score for different groups and different health outcomes and have demonstrated that failure to obey the NSTEMI recommendations will contribute to excess mortality rates, there is no randomized trial to examine if the regular usage of the GRACE score improves NSTE-ACS care and prevents subsequent coronary incidents [11, 12].

In the present study, majority of patients were classified into low risk category followed by intermediate and high risk category. A similar study conducted by Shaikh MK et al. in 2014 found most of the patients were in the high risk group (36%) whereas 35% were in the intermediate risk category and 29% were in the low risk category [13]. Clinically significant finding in the present study showed that GRACE score has good accuracy for predication against optimal cutoff of in-hospital mortality (AUC = 0.80, sensitivity = 93.8% and specificity = 53.2%) and 6 months mortality (AUC = 0.792, sensitivity = 80% and specificity = 70.8%) in patients with NSTE-ACS. Further, our findings showed that in-hospital and 6 months mortality was significantly higher in the high risk category as compared to the moderate risk category whereas no in-hospital mortality was observed in the low risk group and one patient died at 6 months in the low risk category, while Shaikh MK et al. also found similar results and found good discrimination value for GRACE score (AUC = 0.80) and in their study 8.4% of the patients in high risk group died during the hospital stay [13]. Abu-Assi et al. conducted study at Spain in 2010 found 61.2% of the patients admitted in hospital for NSTEMI. Furthermore, GRACE score had excellent discrimination accuracy (AUC = 0.86) for prediction of 6 months mortality [14]. In another study by Thalib L et al. in 6 Arabian Gulf countries for NSTE-ACS found good discrimination value for 6 months GRACE score as AUC = 70.7% for prediction of 1-year mortality [15]. Similarly, Bradshaw et al. also observed good discrimination capacity of GRACE score with c-statistics as 0.80 [16]. In another study by Elbarouni et al., it was observed that the GRACE score is an accurate and powerful tool for the prediction of adverse events across the wide range of ACS patients in Canada [17].

In the current study, after adjustment in the multivariate model, the risk of in-hospital mortality was significantly higher in females, patients with diabetes, and patients who had family history of coronary artery diseases and GRACE score was significant independent predictor of in-hospital mortality, while 6-month mortality was associated with increased age. A similar study by Abu-Assi et al. showed 6-month GRACE risk score was an independent risk factor of 30-day events [14]. A study by Granger et al. found in the multivariate model that in-hospital mortality was higher among patients with diabetes and hypertension; additionally, GRACE risk score was an independent risk for in-hospital mortality [18], whereas in another study, after adjustment for age, risk of in-hospital mortality inflated by 3.5% every year of age. Additionally the odds of in-hospital mortality were higher in females as compared, while ACS patients with diabetes had worse outcomes [19].

The current study’s few drawbacks are that it was a single-center study based on a smaller sample size and the findings lack generalizability for non-tertiary care hospitals and distinct communities. Another drawback of any risk stratification is that it does not inherently accurately estimate the risk of individual person, although it differentiates well between different risk groups. In order to improve the adequacy of the results, prospective multi-center studies of a greater sample size should be carried out.

## Conclusion

GRACE risk score has good predictive value for the prediction of in-hospital mortality and 6 months mortality among patients with NSTE-ACS. Hence, it is useful to include the GRACE scoring for ACS care reports. It might help in contributing to the assessment of therapy outcomes and care of patient.

## Data Availability

Data and material will be available upon request.

## Abbreviations

ACS: Acute coronary syndrome
NSTE-ACS: Non-ST elevation acute coronary syndrome
GRACE: Global Registry of Acute Coronary Events
TIMI: Thrombolysis in myocardial infarction
ROC: Receiver operating characteristic
AUC: Area under the curve
